# Single-Chain Variable Fragment Fusion Proteins for Targeted Delivery and Therapy

**DOI:** 10.3390/ph19081218

**Published:** 2026-08-03

**Authors:** Luona Yang, Yuan Yin, Xinli Liu, Bin Guo

**Affiliations:** Department of Pharmacological and Pharmaceutical Sciences, University of Houston, Houston, TX 77204, USA; lyang40@cougarnet.uh.edu (L.Y.); yyin22@cougarnet.uh.edu (Y.Y.)

**Keywords:** scFv, fusion proteins, targeted therapy, cancer, neurodegeneration, antibody engineering, BBB

## Abstract

A single-chain variable fragment (scFv) is an engineered antibody derivative that retains antigen-binding specificity while having a much smaller size than an antibody, improved tissue penetration, and enhanced versatility for genetic manipulation. When an scFv is fused with diverse protein payloads, multifunctional biologics can be created for targeted delivery and therapy. Over the past decade, scFv fusion proteins have gained significant traction in oncology, where they have been incorporated into immunotoxins, immunocytokines, bispecific antibodies, Chimeric Antigen Receptor (CAR)-T cells constructs, and immune cell engagers. In addition, advances in blood–brain barrier (BBB)-targeting strategies have enabled the exploration of scFv-based therapeutics for neurodegenerative diseases, including Alzheimer’s disease and Parkinson’s disease. Despite promising preclinical and clinical outcomes, challenges such as structural instability, short half-life, immunogenicity, and manufacturing complexity remain. This review provides an in-depth and up-to-date overview of scFv fusion protein engineering and its therapeutic applications in cancer and neurodegenerative disorders. We also highlight the clinical translations and design principles of scFv fusion proteins.

## 1. Introduction

Targeted therapeutics have fundamentally reshaped disease treatment paradigms, particularly in oncology and chronic neurological disorders. Monoclonal antibodies (mAbs) have been at the forefront of this revolution [1]. Despite their clinical success, mAbs possess inherent structural constraints that limit their efficacy in certain disease settings [2]. With a molecular weight of approximately 150 kDa, these large proteins often struggle to penetrate the dense extracellular matrix of solid tumors [3]. Furthermore, their bulky size and lack of specific transport mechanisms render them largely incapable of crossing various biological barriers such as the blood–brain barrier (BBB), blood–retinal barrier (BRB), and mucosal barrier, leaving many pathologies difficult to reach [4,5]. These physiological hurdles have necessitated a move toward smaller, more agile antibody-based scaffolds that maintain high specificity while offering superior pharmacokinetic properties and the potential to penetrate deep into diseased tissues.

**Figure 1:**
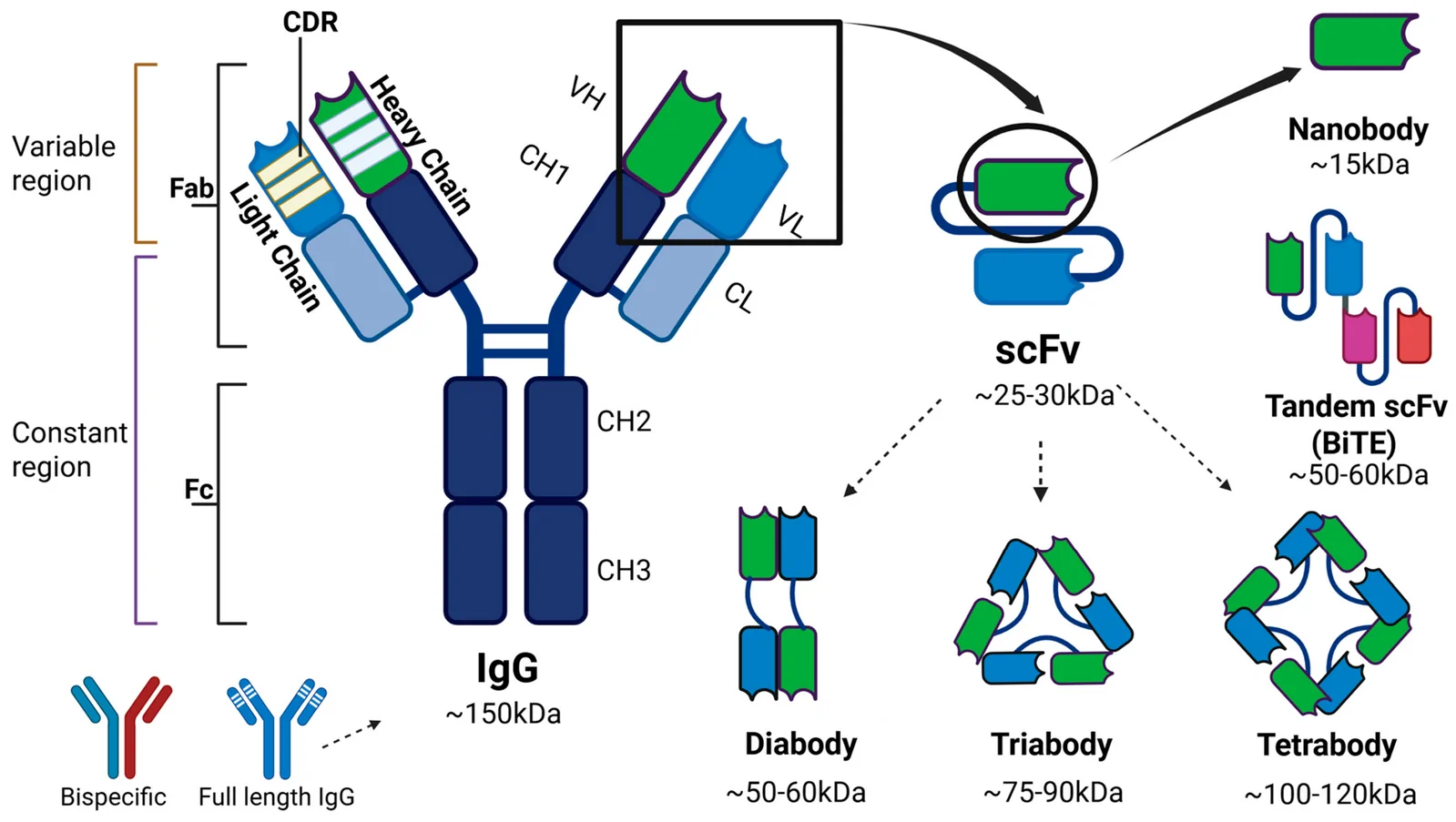
Structural schematic of an IgG antibody, antibody fragments, and its engineered derivatives. This diagram illustrates the relationship between IgG molecule, bispecific antibody, Fab, scFv, nanobody, multivalent scFv formats, and tandem scFv such as BiTE (bispecific T-cell engager) produced through protein engineering. Created in BioRender. Liu, X. (2026) https://BioRender.com/kbe69jk.

The single-chain variable fragment (scFv) represents one of the most successful antibody fragment formats. First described in 1988 [6,7], scFv is a streamlined construct composed of the variable heavy (V_H_) and variable light (V_L_) domains of an antibody (Figure 1), which are joined by a flexible peptide linker (typically a 15–20 amino acid sequence enriched in glycine and serine residues). This elegant design reduces the molecular weight to approximately 25–30 kDa (roughly one-fifth the size of a full-length mAb) while retaining the critical antigen-binding affinity [8]. This drastic reduction in mass facilitates improved diffusion into dense tumor masses and enhances the potential for crossing restrictive physiological barriers. Beyond their structural advantages, scFvs can be expressed efficiently in microbial systems such as *Escherichia coli*. This makes them a cost-effective and versatile platform for developing multispecific proteins, radioimmunoconjugates, and the targeting components of Chimeric Antigen Receptor (CAR)-T cell therapies [9,10]. scFvs can be further engineered by multimerization technology to produce dimers (diabodies, ∼50–60 kDa), trimers (triabodies, ∼75–90 kDa), and tetramers (tetrabodies, ~100–120 kDa). Furthermore, the modular architecture of scFvs enables tandem assembly of two antigen-binding units, as exemplified by bispecific T-cell engagers (BiTEs), in which two scFvs are linked to simultaneously target a tumor-associated antigen and immune cell. A nanobody (or single-domain antibody) is a 15 kDa antibody fragment derived from naturally occurring heavy-chain-only antibodies found in camelids. It represents the smallest antigen-binding entity (Figure 1). Although full-length IgG monoclonal antibodies remain the dominant class of therapeutic biologics, the development of antibody fragments and engineered multispecific formats has expanded the range of available targeting strategies. Among these platforms, scFvs offer a unique combination of small size, modularity, and fusion compatibility, making them particularly attractive for constructing next-generation therapeutics. A comparison of the classic antibody, bispecific antibody, and antibody-fragment platforms is presented in Table 1.

The compact and stable nature of the scFv format enables seamless genetic fusion with a diverse range of functional domains. By tethering an scFv to toxins, cytokines, or enzymes, researchers can engineer multifunctional proteins that act as precision weapons. In oncology, for instance, scFvs are fused to potent cytotoxic agents to create immunotoxins that deliver a lethal attack directly to malignant cells while sparing healthy tissue [11]. Similarly, their integration into CAR-T cell constructs has revolutionized immunotherapy, providing the targeting ligands that allow T cells to recognize specific tumor markers [12]. This modularity also facilitates the creation of bispecific T-cell engagers (BiTEs), which bridge the gap between a cancer cell and a cytotoxic T cell to force an immune synapse [12,13]. Beyond tumor cell killing, these fusion proteins are vital for navigating the complex microenvironments of neurodegenerative disorders and chronic inflammation. In the central nervous system, scFvs can be fused to transport vehicles that hijack receptor-mediated transcytosis pathways, potentially ferrying therapeutic enzymes or protein-aggregation inhibitors across the BBB [14,15].

This review presents a comprehensive analysis of scFv fusion proteins, covering structural design, engineering strategies, therapeutic applications, and emerging technologies shaping the field. We highlight the common engineering principles that govern the success of scFv fusion proteins, such as linker design, stability optimization, half-life extension, immunogenicity reduction, and BBB transport. We also examine both clinically approved therapies and advanced clinical-stage candidates of scFv fusion proteins in oncology and neurological diseases. Beyond soluble fusion proteins, scFvs have been extensively utilized as targeting modules in CAR-T or CAR-NK cell therapies, targeted liposomes, extracellular vesicles, and nanoparticle-based drug delivery systems. Owing to their small size, high specificity, and ease of genetic engineering, scFvs enable selective recognition of disease-associated antigens and enhance targeted delivery of therapeutic payloads. These applications represent a rapidly expanding field that extends beyond the scope of the present review [12,16].

## 2. Structure and Engineering of scFv Fusion Proteins

### 2.1. Molecular Architecture

**Figure 2:**
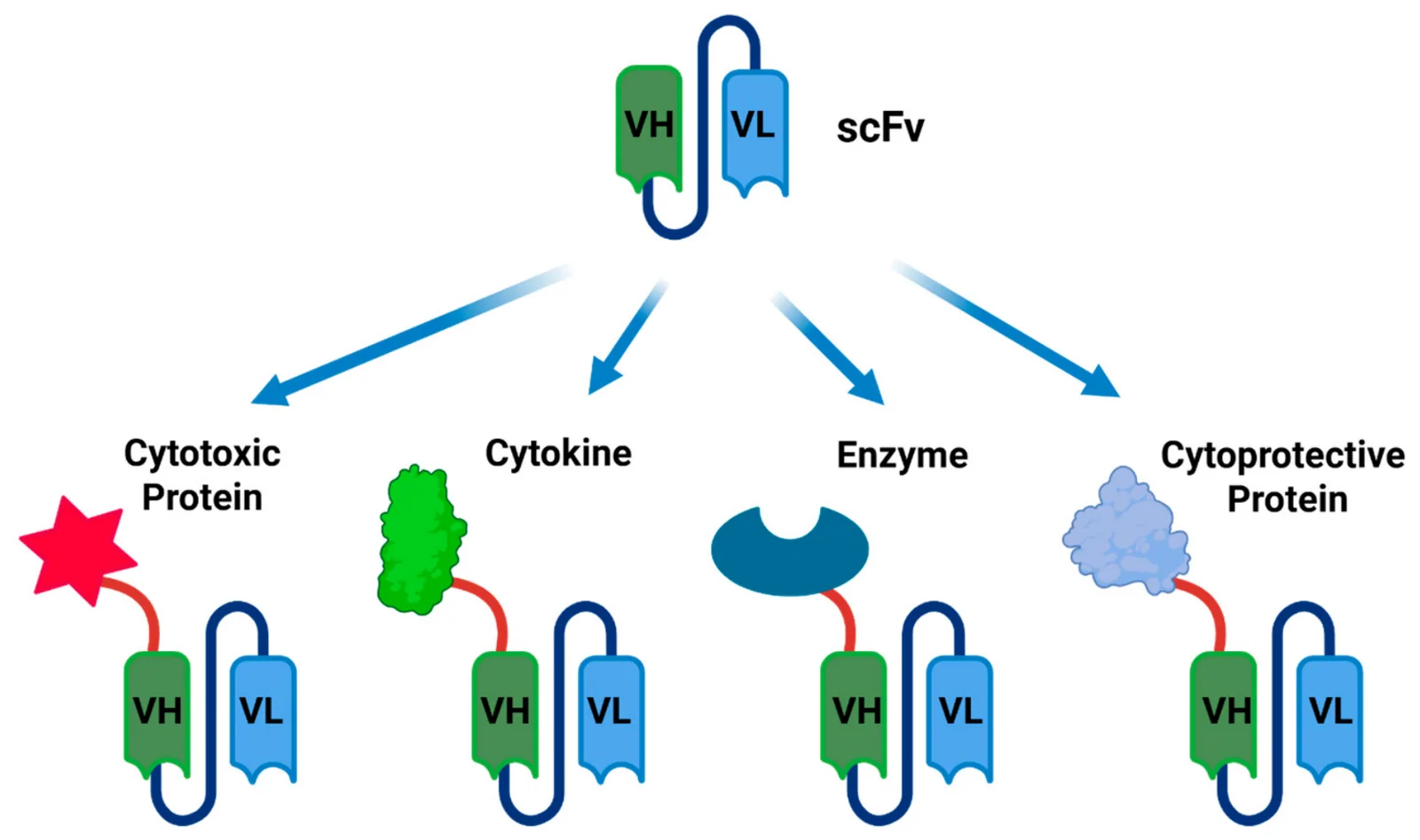
Structural diagram of representative scFv fusion protein formats. Variable heavy (V_H_) and variable light (V_L_) domains are connected by a flexible linker. The scFv can be fused with cytotoxic proteins, cytokines, enzymes, or cytoprotective proteins. Created in BioRender. Guo, B. (2026) https://BioRender.com/qfrg891.

The molecular architecture of an scFv consists of the V_H_ and V_L_ domains of an antibody, which are tethered together by a flexible peptide linker (Figure 1 and Figure 2) [7]. Unlike the parent IgG molecule, which relies on constant domains and inter-chain disulfide bonds for stability, the scFv maintains its structural integrity through the delicate balance of this linker and the intrinsic affinity between the two variable domains [10]. The design of the linker is a critical determinant of the molecule’s success in terms of folding, specificity, and solubility. The most prevalent sequence is the (Gly4Ser)3 motif, a 15-amino-acid chain engineered for high flexibility, reduced steric constraints between domains, and low immunogenicity [17,18]. Glycine provides the necessary conformational freedom, allowing the V_H_ and V_L_ domains to rotate and find their optimal orientation. Serine contributes to solubility, preventing the linker from collapsing into the hydrophobic pockets of the domains. The length of this linker is paramount; a 15-residue span is generally considered the gold standard, providing roughly 3.5 nm of reach [19]. This is sufficient to allow the domains to pair non-covalently without imposing steric strain [20].

Structural integrity hinges on the precise non-covalent pairing of the V_H_ and V_L_ domains. Together, they form the antigen-binding site, or paratope, which is defined by six hypervariable loops known as complementarity-determining regions (CDRs) [21,22]. These loops (three from each domain) can collectively interact with the antigen (Figure 1). Recent advances have highlighted that domain orientation (whether the construct is arranged as V_H_-linker-V_L_ or V_L_-linker-V_H_) is not merely a cosmetic choice. The orientation can significantly dictate the expression yield in microbial hosts and the final binding affinity (K_D_) [23,24]. Improperly folded scFvs are prone to exposing hydrophobic interior residues, leading to irreversible aggregation and loss of function.

### 2.2. Fusion Modalities

scFvs can be fused with various functional modules (Figure 2). For example, scFvs can serve as the targeting modules for highly potent immunotoxins (Figure 2, cytotoxic protein). By genetically fusing an scFv to bacterial-derived toxins, most notably Pseudomonas exotoxin A or diphtheria toxin, engineers have created fusion proteins that deliver lethal cargo with precision [25,26]. The scFv identifies specific surface antigens on malignant cells, triggering receptor-mediated endocytosis. Once internalized, the toxin moiety escapes the endosome and enters the cytosol, where it enzymatically halts protein synthesis by ADP-ribosylating elongation factor 2 [27]. This targeted approach bypasses the systemic toxicity associated with traditional chemotherapy, as the toxin remains inert until it is delivered directly into the cytoplasm of the target cell. Current research focuses on minimizing the inherent immunogenicity of these foreign protein domains to ensure that they can be administered over multiple cycles without being neutralized by the patient’s immune system [28].

Beyond direct cell killing, scFvs are frequently employed as immunocytokines (Figure 2, cytokine), where they are fused to potent signaling molecules like Interleukin-2 (IL-2) [29,30] or Interferon-alpha (IFN-α) [31]. This architectural fusion transforms the systemic administration of cytokines (which is often limited by severe vascular leak syndrome and inflammatory toxicity) into a localized immunotherapy. The scFv moiety delivers the cytokine to the tumor microenvironment (TME), dramatically increasing the local concentration of the signaling molecule. This localized enrichment facilitates the activation of tumor-infiltrating lymphocytes (TILs) and natural killer (NK) cells precisely where they are needed most [32,33]. By tethering cytokines to the site of the disease, we can achieve the desired therapeutic threshold at much lower systemic doses, effectively widening the therapeutic window and reducing the cytokine storm effects that have historically plagued non-targeted cytokine therapies in oncology and chronic viral infections [34].

The modularity of the scFv scaffold is also harnessed in antibody-directed enzyme prodrug therapy (ADEPT) [35] (Figure 2, enzyme), where the fragment is fused to an enzyme such as β-lactamase or carboxypeptidase [36,37]. This dual-function molecule acts as a localized chemical factory; the scFv first navigates to the tumor mass, after which a non-toxic prodrug is administered systemically. The tethered enzyme then catalyzes the conversion of this inactive precursor into a highly cytotoxic drug only within the immediate vicinity of the tumor. This spatial control allows for the generation of extremely high concentrations of the active drug at the tumor site while maintaining negligible levels in healthy tissues [38]. The enzymatically released drug can often diffuse into nearby tumor cells that may not even express the target antigen, ensuring more complete eradication of heterogeneous tumor masses.

The scFv scaffold has been used to mediate delivery of a neuroprotective anti-amyloid therapeutic antibody (Figure 2, protective protein). Using scFvs to deliver protein therapeutics across the BBB represents a frontier in treating neurodegenerative diseases. The BBB is notoriously restrictive, excluding the majority of small molecules and almost all large biologics [39]. To bypass this, scFvs are engineered to hijack endogenous receptor-mediated transcytosis pathways of the BBB. By targeting receptors highly expressed on brain endothelial cells, most commonly the transferrin receptor (TfR) [40], the scFv acts as a transport vehicle. A therapeutic protein cargo can be fused with the scFv, which binds to the receptor to trigger transcytosis [41], and allows the vesicle to transport the cargo into the brain. An anti-TfR scFv was fused to the anti-Aβ antibody mAb158 to enhance BBB transport and increase brain exposure, resulting in improved reductions in soluble Aβ protofibrils in Alzheimer’s disease models [42]. In another report, the anti-TfR scFv was fused to the anti-α-synuclein antibody RmAb38E2-scFv8D3 to enhance brain delivery, reduce pathogenic α-synuclein oligomer levels, and improve microglial activation [43]. Therapeutic neuroprotective enzymes have also been delivered using TfR-mediated BBB transport strategies, as exemplified by pabinafusp alfa (IZCARGO^®^), an anti-TfR antibody–iduronate-2-sulfatase (IDS) fusion protein approved in Japan for the treatment of both physical and neurological symptoms of Mucopolysaccharidosis type II (Hunter syndrome) [44]. These studies highlight the versatility of scFv-based fusion proteins as delivery vehicles for biologics that exert neuroprotective effects.

### 2.3. Expression Platforms

scFv fusion proteins can be expressed and purified using standard techniques for recombinant protein preparations. The selection of an appropriate expression system is important in the development of scFv fusion proteins, as it dictates both the functional integrity of the molecule and the economic feasibility of production. *E. coli* remains the workhorse for initial screening and large-scale production due to its rapid growth kinetics and relatively low cost [45]. This prokaryotic system can generate high yields of protein; however, it often struggles with the complex architecture of scFv fusions. Because *E. coli* lacks the machinery for post-translational modifications, proteins frequently accumulate in insoluble inclusion bodies [46]. This necessitates difficult refolding processes to restore biological activity, which can be inconsistent, significantly lowering the yield of functional proteins.

To bridge the gap between bacterial efficiency and complex folding requirements, researchers often turn to yeast expression systems [47]. Yeast offers the advantage of being an eukaryotic organism that can secrete proteins directly into the culture medium, simplifying the purification process. It provides superior disulfide bond formation compared to bacteria, which is essential for stabilizing the V_H_ and V_L_ domains of the scFv [48,49,50].

For clinical applications where native human-like folding and precise post-translational modifications (PTMs) are required, mammalian cell lines such as Chinese Hamster Ovary (CHO) cells are the gold standard [51]. These cells ensure that the scFv fusion proteins are glycosylated and folded in a manner nearly identical to that of natural human antibodies, maximizing safety and efficacy [52]. Despite these biological advantages, mammalian systems are technically demanding, requiring expensive growth media and sophisticated bioreactors, which drives up the overall cost.

Beyond traditional cell-based platforms, cell-free protein synthesis (CFPS) provides a cost-effective strategy for scFv fusion protein production [53,54]. CFPS bypasses the constraints of cell viability by utilizing crude cellular extracts (containing all the necessary transcriptional and translational machinery) to produce proteins in a controlled chemical environment. This allows for the rapid production of toxic payloads that would otherwise kill a living host cell.

## 3. Applications for Cancer Therapy

### 3.1. Targeted Cytotoxicity

**Figure 3:**
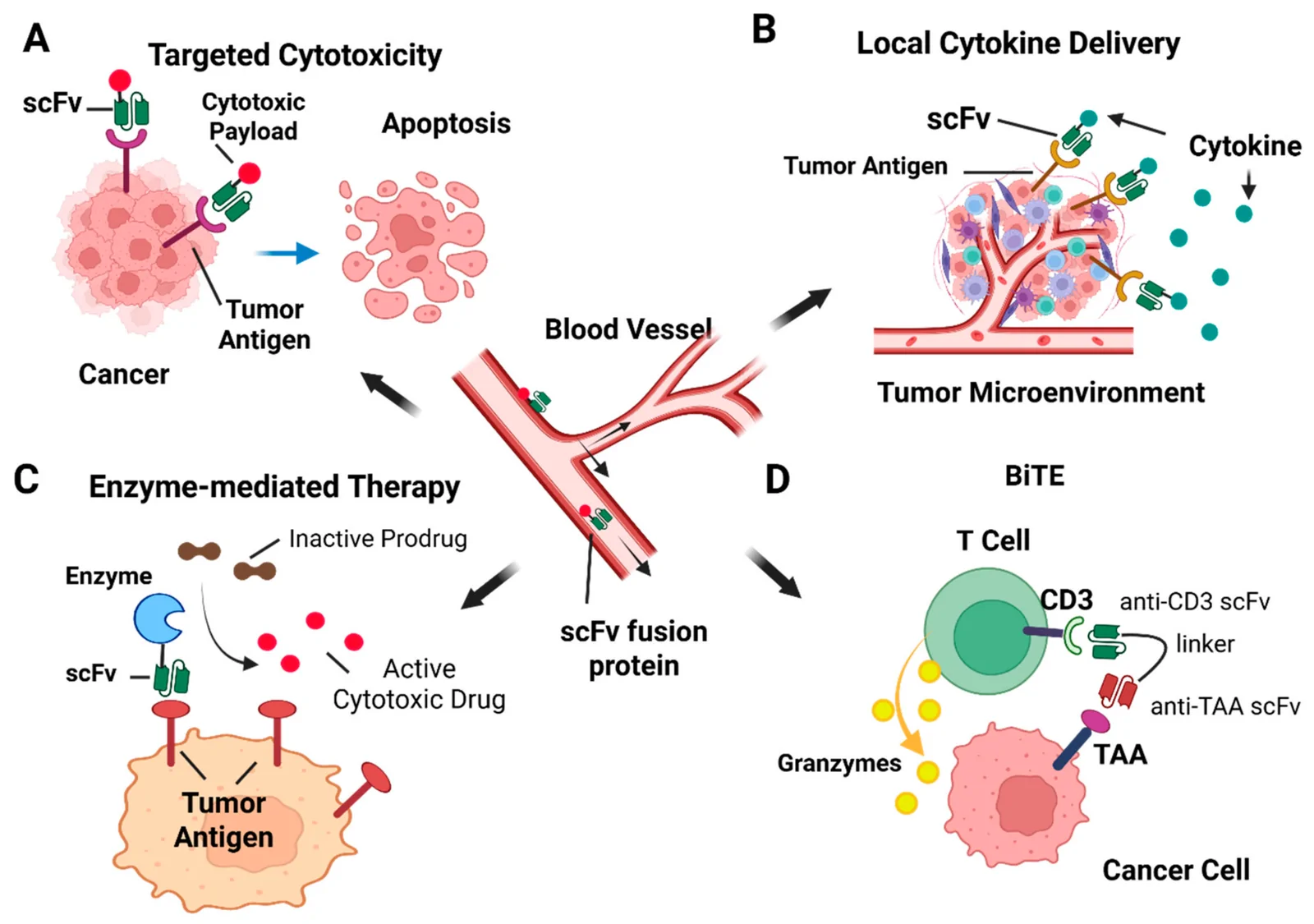
Applications of scFv fusion proteins. (**A**). Proapoptotic proteins or protein synthesis inhibitors can be fused with scFvs for targeted delivery to tumors and can induce apoptosis. (**B**). Cytokines can be delivered to the tumor microenvironment by the scFv fusion to increase the antitumor immune response. (**C**). In ADEPT, enzymes can be delivered to the tumor sites by the scFv fusion to activate prodrugs. (**D**). BiTEs use two scFvs linked together to engage T cells to kill cancer cells. ADEPT: antibody-directed enzyme prodrug therapy. TAA: tumor-associated antigen. Created in BioRender. Guo, B. (2026) https://BioRender.com/j8nrr2v.

When fused with a lethal payload, an scFv acts as a guided missile, delivering the attack directly to malignant cells while sparing healthy tissues (Figure 3A). The mechanism begins with high-affinity binding to specific cell-surface antigens (such as HER2 in breast cancer). Upon binding, the entire complex is typically internalized via receptor-mediated endocytosis. The variety of payloads available for fusion is extensive and is categorized by their mechanism of action. Bacterial or plant toxins, such as *Pseudomonas* exotoxin [55] or ricin [56], are common choices; once they escape the endosome into the cytosol, they catalytically inactivate protein synthesis, leading to rapid cell apoptosis. For example, the anti-HER2 scFv was fused to the protein synthesis inhibitor gelonin and inhibited breast cancer xenograft growth in mice [57]. Alternatively, pro-apoptotic proteins like Granzyme B [58] or various caspases [59] can be delivered to directly trigger the cell’s internal self-destruction program. For example, Hutt et al. constructed fusion proteins linking proapoptotic TRAIL to scFvs targeting EGFR or HER3 achieving strong antitumor activity in xenograft tumor models [60]. An anti-HER2 scFv was fused with cytochrome c, which induced apoptosis in HER-2-overexpressing breast cancer cells [61]. An anti-CD22 scFv fused with the proapoptotic protein Bim effectively induced apoptosis in malignant B cells [62]. A dual-target therapeutic system, in which mesenchymal stem cells were engineered to produce and deliver scFv-Fdt-tBid, significantly inhibited prostate cancer growth in vivo [63]. Regardless of the payload, the goal is to induce irreversible cellular damage, which manifests as programmed apoptosis or inflammatory necrosis, thus effectively decreasing the tumor mass.

### 3.2. Tumor Microenvironment Targeting

scFv fusion proteins can modulate the tumor microenvironment (TME) by delivering cytokines or checkpoint inhibitors locally (Figure 3B). The therapeutic potential of scFv–cytokine fusions, often termed immunocytokines, lies in their ability to concentrate potent immune-modulating signals directly within the TME [64,65]. Systemic administration of cytokines like IL-2, IL-12, or TNF-α is frequently limited by severe cytokine storm toxicities [66,67]. However, by anchoring these molecules to an scFv that targets tumor-associated antigens (TAAs) such as carcinoembryonic antigen (CEA) [68] or stromal markers like Fibroblast Activation Protein (FAP) [69], the cytokine dose is sequestered at the tumor site. This local enrichment transforms the TME from an “immunologically cold” (suppressive) state to a “hot” (active) state, stimulating the infiltration and activation of natural killer (NK) cells and cytotoxic T lymphocytes (CTLs) [70]. For example, Halin et al. constructed a fusion protein composed of IL-12 and an scFv targeting the ED-B domain of fibronectin, achieving strong antitumor activity in a mouse lung-metastasis model [71]. Xu et al. engineered a fusion protein consisting of scFv targeting CEA and IL-2, which demonstrated excellent antitumor activity in mice bearing MC-38 tumors [68].

Furthermore, scFvs can be engineered to deliver checkpoint inhibitors or costimulatory molecules locally. For example, Tzuri et al. created DuRan-Bis, comprising an scFv-based inhibitor of PD-L1 fused to an scFv-based inhibitor of VEGF, which showed superior inhibition of the proliferation of glioblastoma cells [72]. This targeted approach not only enhances the antitumor immune response but also significantly minimizes immune-related adverse events (irAEs) in healthy organs [73].

### 3.3. Enzyme-Mediated Therapy

scFv–enzyme fusions enable ADEPT by converting inactive prodrugs into active cytotoxic agents at target sites (Figure 3C). ADEPT is a sophisticated approach to cancer treatment, designed to maximize the therapeutic index of highly potent cytotoxic agents. In this mechanism, an scFv–enzyme fusion protein is administered first. The scFv acts as a targeting module, binding to specific antigens on the tumor cell surface and anchoring the enzyme moiety within the tumor. After the fusion protein has cleared from the systemic circulation, a non-toxic prodrug is administered. Upon reaching the tumor, the tethered enzyme converts the prodrug into its active, lethal form. This localized conversion creates a high concentration of the drug exactly where it is needed, effectively sparing healthy tissues from systemic toxicity. For example, an anti-CEA scFv was fused to the bacterial enzyme carboxypeptidase G2 (CPG2), which cleaves mustard-based prodrugs [36,74]. The active mustard drug then cross-links DNA in tumor cells, inducing rapid cell death. An anti-TAG-72 scFv was fused to β-lactamase, which can activate the novel prodrug GC-Mel, resulting in significant efficacy in a colorectal xenograft tumor model [75]. An anti-A33 scFv was fused with Cytosine Deaminase (CD), which converts 5-fluorocytosine (5-FC) into the potent chemotherapeutic 5-fluorouracil (5-FU), a strategy often employed in treating colon cancer [76].

### 3.4. Bispecific T-Cell Engagers (BiTEs)

A BiTE is composed of two scFvs [anti-CD3 and antitumor-associated antigen (TAA)] joined by a short flexible linker [77]. The mechanism of BiTEs (Figure 3D) relies on the simultaneous engagement of T cells and tumor cells via their scFvs. When the anti-TAA scFv anchors to a tumor cell and the anti-CD3 scFv binds to a T cell, it forces the formation of a cytolytic synapse. This interaction triggers T-cell activation, leading to the release of perforins and granzymes that induce apoptosis in target cancer cells [78]. For example, blinatumomab (the first FDA-approved BiTE), targeting CD19 on B-cell precursor acute lymphoblastic leukemia (ALL), has revolutionized the treatment for patients with minimal residual disease [79]. Tebentafusp, a BiTE targeting glycoprotein 100 and CD3 for uveal melanoma, demonstrates that the technology can be applied to the treatment of solid tumors [80].

## 4. Applications for Neurodegenerative Diseases

### 4.1. Blood–Brain Barrier Targeting

**Figure 4:**
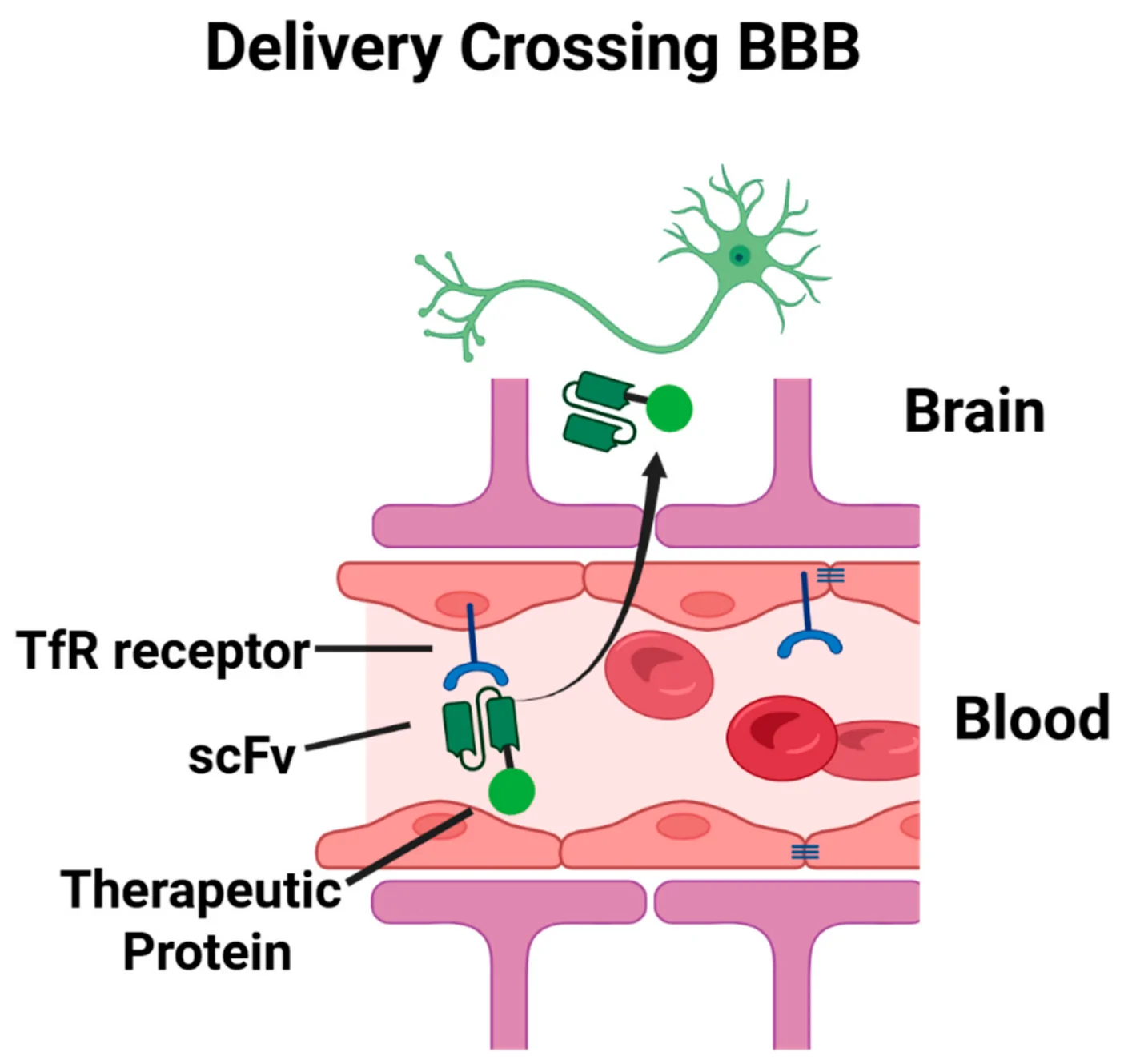
Delivery of therapeutic proteins across the BBB. Therapeutic proteins such as antibodies targeting Aβ can be delivered across the BBB into the brain using the anti-TfR scFv. Created in BioRender. Guo, B. (2026) https://BioRender.com/6vvp5e1.

The BBB is a major obstacle to CNS therapeutics. To overcome this, scFvs are engineered to carry therapeutic proteins across the BBB by targeting endogenous transport systems, specifically receptor-mediated transcytosis (Figure 4). By binding to receptors highly expressed on the brain microvasculature, such as the TfR or the insulin receptor (IR) [81], these scFv constructs hijack the natural cellular machinery intended for essential nutrients. Furthermore, scFv affinity can be finely tuned to ensure that the protein binds to the luminal receptor strongly enough for internalization but is effectively released upon reaching the abluminal (brain) side [81]. For example, Hultqvist et al. engineered a fusion protein consisting of the anti-TfR scFv and the light chains of mAb158 (an antibody for Aβ protofibrils, which are involved in Alzheimer’s disease), resulting in significantly high brain uptake of the fusion protein [42]. It was shown that the anti-TfR antibodies with high affinity for TfR remain associated with the BBB, whereas lower-affinity anti-TfR antibody variants are released from the BBB into the brain, as high-affinity anti-TfR antibodies bind so strongly to the TfR on the luminal side of the BBB that they cannot detach once they reach the abluminal side of the BBB, trapping the antibody at the barrier. Thus, Yu et al. designed a bispecific antibody that binds with low affinity to the TfR and with high affinity to the enzyme β-secretase (which processes amyloid precursor protein into Aβ), resulting in high accumulation in the mouse brain and a greater reduction in brain Aβ after a single systemic dose [82].

Previous reviews have discussed the potential of scFvs as therapeutic agents for neurodegenerative diseases, particularly their ability to target pathogenic protein aggregates and facilitate passive immunotherapy approaches. However, most earlier analyses primarily focused on stand-alone scFvs and antibody fragments [83,84]. Recent advances have expanded the field toward multifunctional fusion proteins incorporating BBB transport modules, therapeutic proteins, enzymes, and multispecific architectures, opening new avenues for harnessing the potential of scFvs in treating neurological diseases.

### 4.2. Alzheimer’s Disease

Alzheimer’s disease (AD) is characterized by the toxic accumulation of Aβ plaques and intracellular tau neurofibrillary tangles [85]. scFv fusion proteins have emerged as potent tools to intervene in AD progression. By targeting the N-terminal or central hydrophobic core of Aβ, scFv constructs can physically interfere with the aggregation process, preventing the formation of toxic oligomers and reducing the overall plaque burden [86]. Because scFvs can be engineered to be bivalent or fused to clearance-promoting moieties, they enhance the brain’s ability to flush these toxins, often by reprogramming microglia [87]. For example, when the Aβ protofibril-selective protein mAb158 was fused with an anti-TfR scFv, Syvänen et al. showed a 40% reduction in soluble Aβ protofibrils in mice [88]. Conversely, Sumbria et al. constructed a fusion protein consisting of an anti-amyloid scFv and the carboxyl terminus of the heavy chain of an mAb against the mouse TfR, achieving 57% and 61% reductions in amyloid plaques in the cortex and hippocampus, respectively [89]. Beyond therapy, these fusions are revolutionizing early diagnostic imaging. By conjugating an anti-Aβ or anti-tau scFv to a contrast agent (such as a radionuclide for PET), clinicians can visualize the molecular pathology of AD years before clinical symptoms manifest [90,91]. This early detection is critical for the success of disease-modifying therapies.

### 4.3. Parkinson’s Disease

In Parkinson’s disease (PD), the primary pathological driver is the misfolding and aggregation of α-synuclein, which forms insoluble Lewy bodies that lead to the death of dopaminergic neurons [92]. scFv-based constructs can be specifically designed to target the most toxic species of α-synuclein: the soluble oligomers and protofibrils [93]. These fusion proteins can be engineered to recognize unique conformational epitopes that are only present in the diseased protein, leaving normal, healthy α-synuclein untouched. This selectivity is vital for maintaining normal synaptic function while simultaneously promoting the clearance of pathological aggregates. For example, an anti-TfR scFv was fused with the anti-α-synuclein oligomer antibody RmAb_3_8E_2_ and this fusion protein reduced brain α-synuclein oligomer levels and increased microglial activation in transgenic L61 mice [43]. An et al. engineered a bispecific antibody composed of an α-synuclein-binding immunoglobulin and an insulin-like growth factor receptor 1-binding scFv, which binds to α-synuclein aggregates and prevents their seeding capacity in vitro and in vivo [94]. The delivery of these scFvs into the CNS leads to a significant reduction in neurotoxicity and neuroinflammation. By neutralizing extracellular α-synuclein, scFv fusions prevent the protein from being taken up by neighboring healthy neurons, thereby breaking the cycle of transmission [95].

## 5. Clinical Translation

### 5.1. Approved Therapies

The clinical landscape for scFv-based therapies has evolved from experimental concepts to robust pipelines of clinical-stage investigational medicines or clinically approved medicines, fundamentally changing the treatment paradigm for cancer. The representative clinically approved or investigational scFv fusion drugs are listed in Table 2. Blinatumomab (Blincyto) is a hallmark success, representing the BiTE class. It consists of two scFvs targeting CD19 on B cells and CD3 on T cells. By physically bridging these cells, it forces the immune system to recognize and destroy leukemic cells, specifically in patients with B-cell precursor ALL [96]. In the realm of immunotoxins, the engineering of bacterial toxins has led to significant clinical breakthroughs. Moxetumomab pasudotox, which utilizes an anti-CD22 scFv fused to the PE38 payload, is a prime example [97]. This drug has been approved for relapsed/refractory hairy cell leukemia, demonstrating that scFv can effectively deliver lethal payloads to resistant cancer cells.

### 5.2. Ongoing Clinical Trials

Recently, there has been a significant increase in clinical trials exploring scFv fusion proteins, with a strong focus on solid tumors and neurological disorders. For example, an EGFR-targeted immunotoxin consisting of a D2C7 scFv and PE38 is in a Phase I/II study (NCT02303678) for adults with recurrent malignant glioma [98]. VB4-845 is an scFv fusion protein that targets the epithelial cell adhesion molecule (EpCAM) and carries Pseudomonas exotoxin [99]. It has been evaluated in clinical trials for head and neck cancer and bladder cancer. Perhaps the most exciting frontier in current clinical trials is the development of BBB-penetrating biologics for Alzheimer’s disease. Trontinemab, which is specifically designed to cross the BBB via receptor-mediated transcytosis, is a bispecific monoclonal antibody that binds bivalently to Aβ plaques and monovalently to the human TfR (2 + 1 format) [100]. The monovalent TfR-binding domain rather than the bivalent domain was designed to prevent high-affinity TfR clustering, avoid intracellular mistrafficking, and ensure that trontinemab successfully exits endothelial cells into the brain parenchyma. Phase Ib/IIa results of trontinemab showed rapid, high-level amyloid plaque clearance with low amyloid-related imaging abnormalities (ARIAs; brain swelling/bleeding) rates. These trials aim to achieve significant amyloid and tau clearance at lower systemic doses, potentially reducing the incidence of ARIAs and providing a safer and more effective strategy for treating neurodegeneration.

**Table 2:** Clinically approved and clinical-stage scFv fusion proteins for cancer and neurodegenerative diseases.

Therapeutic Area	Drug/Construct	Target	Payload/Format	Status	Ref.
Oncology	Blinatumomab (Blincyto)	CD19/CD3	BiTE (scFv-scFv)	Approved (B-cell precursor ALL)	[96]
Oncology	Moxetumomab pasudotox (Lumoxiti)	CD22	PE38 immunotoxin	Approved (hairy cell leukemia)	[97]
Oncology	Tarlatamab-dlle (Imdelltra)	DLL3/CD3	scFv-based BiTE T-cell engager	Approved (small-cell lung cancer)	[101]
Oncology	Tebentafusp (Kimmtrak)	gp100 peptide-HLA-A*02:01/CD3	T-cell receptor fused to an anti-CD3 scFv	Approved (uveal melanoma)	[102]
Oncology	Vicinium (VB4-845)	EpCAM	Pseudomonas exotoxin	Phase III (bladder cancer)	[99]
Oncology	L19TNF	Fibronectin	TNF-α immunocytokine	Phase III (brain tumor)	[103]
Oncology	Darleukin	Fibronectin	IL-2 immunocytokine	Phase III (melanoma)	[104]
Oncology	D2C7-IT	EGFRwt/EGFRvIII	scFv-PE38KDEL immunotoxin	Phase I/II (brain cancer)	[105]
Neurology	Pabinafusp alfa (IZCARGO)	TfR/IDS	Anti-TfR antibody–IDS fusion protein	Approved in Japan (Hunter syndrome)	[44]
Neuro-degeneration	Trontinemab	Aβ/TfR	bispecific anti-Aβ/anti-TfR	Phase III (Alzheimer’s disease)	[100]

## 6. Challenges and Optimizations

### 6.1. Stability Engineering

scFvs are known to be less stable than their parent monoclonal antibodies, often prone to unfolding or aggregation due to the exposure of hydrophobic patches that were originally hidden by constant domains. Stability engineering addresses this issue via disulfide bond stabilization by introducing disulfide bonds between the scFv linker and the two variable domains [106]. Stapling increased thermal stability (Tm) and reduced aggregation while retaining binding affinity and functionality. Beyond physical stapling, framework optimization involves swapping unstable protein sequences for consensus frameworks known for their thermal and chemical robustness [107].

Optimization of scFv linker length and composition can favor correct intramolecular V_H_-V_L_ pairing and significantly improve protein stability, solubility, expression yield, and antigen-binding activity while minimizing aggregation and misfolding. Excessively short linkers may promote intermolecular pairing and high-order oligomerization (like diabodies), while overly long flexible linkers are more exposed to the solvent and vulnerable to protease attacks, leading to product degradation [108]. Two anti-CD22 scFvs with short (GGGGS) or long ((GGGGS)_4_) linkers were compared using molecular dynamics simulations. The short-linker scFv exhibited higher affinity (Kd = 5.1 nM vs. 42.1 nM), slower dissociation, and greater avidity for CD22. While both scFvs are mostly monomeric, transient dimers were seen in the short-linker scFv [109]. More recently, semi-rigid (proline-rich regions), helical (often EAAAK repeats), and protease-resistant linkers have been explored to further enhance conformational stability and preserve functional activity in complex scFv fusion constructs [110,111,112]. Therefore, rational linker engineering has emerged as a key strategy for improving the developability, manufacturability, and therapeutic performance of scFv fusion proteins.

### 6.2. Improving Pharmacokinetics

Conventional naked, unfused scFvs are relatively small proteins (~25–30 kDa); they are subject to rapid renal filtration and clearance, often resulting in plasma half-lives of less than a few hours, which are much shorter than those of intact IgG, which extends its circulation half-life through neonatal Fc receptor (FcRn)-mediated recycling [113]. While the small size of scFvs enhances tissue penetration and tumor diffusion, it can also reduce systemic exposure and therapeutic duration, necessitating frequent dosing. This short half-life of naked scFvs may be adequate for imaging but requires further optimization for therapeutic use. Fusion protein architecture, linker design, target-mediated drug disposition, and payload-specific properties can significantly influence the biodistribution and clearance of scFv fusion proteins. Several engineering strategies have been developed to improve the pharmacokinetic properties of scFv constructs. Multivalent scFv formats, including diabodies, triabodies, disulfide-linked scFv dimers, and tandem or fusion-linked scFvs constructs, can increase the overall molecular size beyond the renal filtration cutoff (~60 kDa), prolong circulation half-life, and enhance functional affinity through avidity effects, thereby improving in vivo targeting. Alternative approaches include albumin-binding domains, albumin fusion, Fc fusion, and PEGylation technologies, which increase the hydrodynamic radius of scFv fusion proteins and reduce renal clearance [114,115,116]. Charge optimization and surface engineering of scFv fusion proteins such as lowering elevated isoelectric points or removing patches of positive charges or excessive hydrophobicity could reduce nonspecific tissue accumulation and off-target clearance mechanisms [117]. In BBB-targeting biologics, receptor-mediated transport systems such as TfR-binding shuttles require careful optimization of affinity and pharmacokinetics because excessively high receptor affinity can promote endothelial retention and lysosomal degradation, whereas moderate affinity facilitates efficient transcytosis into the brain parenchyma. Recent advances in protein engineering, computational design, and PK/PD modeling have enabled a more rational balance between tissue penetration, systemic exposure, binding affinity, and therapeutic efficacy, thereby improving the clinical utility of next-generation scFv fusion proteins.

### 6.3. Immunogenicity Reduction

scFv fusion protein therapy can fail in the clinic if the patient’s immune system recognizes it as foreign. Immunogenicity reduction is centered on the process of humanization [118], where the non-human CDRs of a mouse-derived scFv are grafted onto a human scaffold. Furthermore, epitope masking or de-immunization is used to identify and mutate specific peptides that are likely presented by MHC-II molecules to T cells [119]. By altering these residues, the visibility of scFv to the immune system is lowered, reducing the risk of anti-drug antibodies (ADAs). ADAs are a major concern because they can neutralize the drug’s activity or cause dangerous allergic reactions. Successfully reducing immunogenicity ensures that the scFv fusion protein can be administered repeatedly over long treatment cycles, which is particularly critical for chronic conditions like Alzheimer’s or Parkinson’s disease.

scFv fusion proteins are an excellent modular engineering platform that bridges antibody specificity with programmable therapeutic functions, enabling next-generation targeted biologics for cancer and CNS diseases. The development of effective scFv fusion proteins requires optimization of multiple interconnected parameters, including antigen selection, affinity, valency, payload composition, pharmacokinetics, tissue penetration, and safety. These factors collectively determine the efficacy, selectivity, and translational potential of the final scFv-based therapeutic constructs. The major design considerations and engineering strategies that guide the rational engineering of scFv fusion proteins for oncology and neurodegenerative disease applications are summarized in Table 3.

## 7. Conclusions

Drug development for oncology and neurodegenerative diseases is challenging with a low success rate. scFv fusion proteins represent a highly adaptable and powerful platform for targeted therapy because of their small size, modularity, and ability to be engineered for diverse biological functions. By genetically linking scFvs with cytokines, toxins, enzymes, cytoprotective proteins, or immune-engaging domains, researchers can create multifunctional therapeutics with improved specificity and reduced off-target toxicity. Advances in protein engineering, linker optimization, and computational modeling have greatly enhanced the stability, affinity, pharmacokinetics, and manufacturability of these molecules. scFv fusion proteins are now being actively explored in cancer immunotherapy, including bispecific T-cell engagers and immunocytokines, as well as in neurodegenerative diseases, through BBB shuttle systems targeting receptors such as the TfR. Ongoing innovations in half-life extension, conditional activation, and AI-guided molecular design are expected to overcome these limitations and accelerate the clinical translation of next-generation scFv-based therapeutics.

## Figures and Tables

**Table 1:** Simplified comparison of scFv, full-length IgG, nanobody, and bispecific antibody platforms.

Feature	Full-Length IgG	scFv	Nanobody	Bispecific Antibody
Molecular size	~150 kDa	~25–30 kDa	~15 kDa, smallest antibody-derived format	~50–200 kDa, depending on format
Structure	VH and VL domains with constant regions and an Fc domain	VH and VL connected by a flexible peptide linker	Single variable heavy-chain domain	Two antigen-binding specificities in IgG-like or fragment-based formats
Target specificity	Single-target	Usually single-target, easily engineered to multivalent or multispecific	Usually single-target, easily engineered to multivalent or multispecific	Dual targets or dual epitopes by design
Tissue penetration	Moderate; limited by large molecular size	High; smaller size supports better diffusion	Very high; efficient diffusion into dense tissue	Variable; moderate for large IgG-like formats
Serum half-life	Long due to FcRn recycling	Short unless half-life extension is engineered	Very short unless half-life extension is engineered	Variable; IgG-like formats are long-acting,
Multispecific engineering	More challenging than fragment formats	Suitable for multivalent and multispecific constructs	Suitable for multivalent and multispecific constructs	Intrinsic feature of the platform
Stability	Generally high	Moderate; can be improved by linker or disulfide engineering	Generally high	Variable; depends strongly on format
Major advantages	Long half-life, high stability, and established clinical platform	Small size, high modularity, and easy fusion with toxins, cytokines, enzymes, immune engagers, or BBB shuttles	Smallest size, high tissue penetration, high stability, and simple production	Simultaneous targeting of two epitopes or antigens
Major limitations	Poor penetration into solid tumors, dense tissue and the CNS	Short half-life, a high tendency to aggregate, need stability, PK optimization	Rapid renal clearance and potential immunogenicity if not humanized	Manufacturing complexity

**Table 3:** Design considerations for optimizing scFv fusion proteins for oncology and neurodegenerative diseases.

Design Variable	Key Issues	Biological Impact & Engineering Strategies
scFv affinity	Affinity that is too high can impair tissue penetration or BBB release; affinity that is too low can reduce efficacy	Influences receptor occupancy, internalization, tissue distribution, target retention, BBB release, off-target binding, and the PK/PD relationship
Valency and format	Avidity (monovalent or multivalent) and molecular size can impact specificity, PK, and tissue penetration	Optimize avidity and tissue penetration according to the design mechanism, e.g., tandem scFvs
Linker design	Long vs. short and flexible vs. rigid linkers can impact steric hindrance, conformational stability, and protease stability	Affects folding, binding activity, aggregation, payload accessibility, structural stability, and immunogenicity, e.g., (G4S)n flexible linkers
Payload type	Payload choice dictates delivery approaches. Toxin, cytokine, enzyme, neuroprotective protein, antibody, immune-engaging cargo, etc.	Determines the mechanism of action, therapeutic window, and potential toxicity, e.g., IL-2
Pharmacokinetics	Rapid clearance, insufficient exposure, and balance between exposure and tissue penetration	Half-life extension by Fc fusion, albumin fusion, PEGylation, and charge and sequence optimization
Tissue penetration	Dense extracellular matrix, restricted diffusion, binding-site barrier, the balances among diffusion, receptor binding, and clearance	Affinity-tuned scFv format optimization to influence intratumoral penetration, biodistribution, and off-target nonspecific binding
BBB transport	Poor CNS delivery, endothelial receptor trapping, and peripheral receptor binding	Optimize brain receptor-mediated transcytosis while avoiding receptor trapping, peripheral sink effects, lysosomal degradation to improve CNS drug exposure, e.g., TfR fusion
Immunogenicity	Anti-drug antibody formation, hypersensitivity reactions, payload immunogenicity, and loss of exposure	Enables repeated dosing, chronic administration, and improved clinical tolerability, e.g., humanized scFvs

## Data Availability

No new data were created or analyzed in this study. Data sharing is not applicable to this article.

## Abbreviations

The following abbreviations are used in this manuscript:

BBB	Blood–brain barrier
TfR	Transferrin receptor
scFv	Single-chain variable fragment
mAb	Monoclonal antibody
BiTEs	Bispecific T-cell engagers
CDRs	Complementarity-determining regions
AD	Alzheimer’s disease
TME	Tumor microenvironment
ADEPT	Antibody-directed enzyme prodrug therapy
CFPS	Cell-free protein synthesis
ARIAs	Amyloid-related imaging abnormalities
PD	Parkinson’s disease
CNS	Central nervous system
